# Risk assessment studies on HLA association with drug induced hypersensitivity caused by anti tuberculosis drugs – an *in silico* approach

**DOI:** 10.1186/1471-2334-14-S3-P45

**Published:** 2014-05-27

**Authors:** Prashant Saxena, Jemmy Christy

**Affiliations:** 1 Department of Bioinformatics, Sathyabama University, Chennai-600119, India

## Background

Studies have conferred that there is a strong genetic association between HLA alleles and susceptible drugs leading to hypersensitivity. Patients with HIV have the highest risk of developing active Tuberculosis. So here we studied the genetic predisposition to anti-tuberculosis drugs as a model to study the pathological role of HLA alleles in drug hypersensitivity.

## Method

Here we aimed to find out the anti-tuberculosis drugs like sulfasalazine, allopurinol, streptomycin and oflaxacin as candidate drugs and their binding sites on HLA alleles which are prevalent among Indian population like HLA-A*02:06, HLA-B*57:01, HLA-A*02:01 and HLA-A*02:03 using docking simulation method via Autodock.

## Result

Anti-tuberculosis drugs can bind within the peptide binding grooves of HLA-B*57:01, HLA-A*02:06 and HLA-A*02:03 alleles and thereby alter its specificity. In the docking simulations, the interactions were found between HLA-A*02:01 and sulfasalazine , streptomycin , oflaxacin at Asp54, Ile237, Glu236, Lys267, Glu256, Thr238 And Val255 with -6.43 kcal/mol, -5.86 kcal/mol and -5.83 kcal/mol binding energy. HLA-A*02:06, HLA-B*57:01 and HLA-A*02:03 have interaction with oflaxacin at Arg6, Lys58, Tyr51 and Arg72 with binding energies of -5.72 kcal/mol, -5.79 kcal/mol and -5.15 kcal/mol respectively.

## Conclusion

The ultimate translation of this knowledge of how Anti-tuberculosis drugs interact with HLA would be applicable to preclinical drug screening programs to improve the safety and cost effectiveness of drug design and development.

